# The development and deployment of a field-based loop mediated isothermal amplification assay for virulent *Dichelobacter nodosus* detection on Australian sheep

**DOI:** 10.1371/journal.pone.0204310

**Published:** 2018-09-27

**Authors:** Nickala Best, Brendan Rodoni, Grant Rawlin, Travis Beddoe

**Affiliations:** 1 Department of Animal, Plant and Soil Science and Centre for AgriBioscience (AgriBio), La Trobe University, Bundoora, Melbourne, Victoria, Australia; 2 Department of Economic Development, Jobs, Transport and Resources Centre for AgriBioscience (AgriBio), Victorian Government, Bundoora, Melbourne, Victoria, Australia; University of Helsinki, FINLAND

## Abstract

*Dichelobacter nododus* is the causative agent of footrot, a major disease of sheep that creates welfare concerns and large economic loss. The virulence of *D*. *nododus* depends on the presence of extracellular proteases, AprV2 and AprB2, which differ by one amino acid. Strains possessing AprV2 can cause clinically virulent disease, while AprB2 may cause clinically benign disease. Current methods for detecting *D*. *nodosus* are difficult, laborious and time consuming. New techniques capable of rapidly detecting and typing *D*. *nodosus* are needed to aid control programs. Molecular methods, like real-time polymerase chain reaction (rtPCR) can detect *aprV2* and *aprB2*, however, this assay is not field-deployable and cannot support local decision-making during an outbreak. Here we present a field-based molecular assay for detecting *aprV2*, using loop mediated isothermal amplification (LAMP). The *aprV2* LAMP (VDN LAMP) assay was optimised to reliably detect *aprV2* from laboratory purified genomic (gDNA) of virulent *D*. *nodosus* down to 5x10^-3^ ng μL^-1^, with time to positive (Tp) ≤ 16 minutes, while *aprB2* was unreliably detected at 5 ng μL^-1^ from 16–20 minutes. The use of field collected samples that were rtPCR positive for *aprB2* resulted in no amplification, while *aprV2* positive field samples by VDN LAMP assay are defined as having Tps’ of < 20 minutes and melting temperature between 88.0–88.9°C. When compared to rtPCR, the VDN LAMP was shown to have a diagnostic specificity of 100% and sensitivity of 83.33%. As proof of concept, the VDN LAMP was taken on farm, with all processing occurring in-field. The on farm VDN LAMP successfully detected 91.67% *aprV2* positive samples, no *aprB2* positive samples (n = 9) or *D*. *nodosus* negative (n = 23) samples, with a kappa agreement of ‘almost perfect’ to rtPCR. This highlights the potential of the assay to inform local treatment decisions for management.

## Introduction

Footrot is a bacterial disease of sheep, first recorded 200 years ago [1]. Its primary aetiological agent is *Dichelobacter nodosus*, an aerotolerant anaerobe capable of digesting the hoof material, causing painful lesions and underrun in the sheep foot [2]. Research into footrot and diagnostics in Australia has been conducted since the 1940’s, progressing from histopathology [3], culturing [4], and now to molecular methods, such as real time polymerase chain reaction (rtPCR), for the detection and strain typing of *D*. *nodosus* [5]. Footrot has been traditionally classed into two forms, benign and virulent, based on the infecting *D*. *nodosus* strains’ ability to digest the hoof substrate and the clinical presentation of the disease [6]. An association has been shown between severe lesions and the presence of an extracellular protease, AprV2, which is responsible for the overall elastase activity and development of footrot [7]. Conversely, the benign *D*. *nodosus* protease equivalent, AprB2, has been shown to cause mild lesions [7]. Genomic sequencing of over 100 strains of *D*. *nodosus* has shown two distinct clades, based on the *aprV2/aprB2* protease genes [8], differentiated by dinucleotide polymorphism (TA/CG) at position 661–662. This change results in a single amino acid change (Tyr92Arg), which affects the thermostability and elastase activity of the protease molecules [9, 10].

Diagnosis of clinical benign or virulent forms of footrot helps to identify the cost-benefit to enacting on-farm control or eradication programs, based on production losses and cost of the program [11]. The clinical expression of footrot is reliant on the presence of an infecting *D*. *nodosus* strain, animal genetics, and environmental conditions. Wet and warm conditions, above 10°C, have been reported to be ideal for *D*. *nodosus* to cause disease [1]. An infected sheep may show no or little sign of clinical disease if it has a natural resistance, or if the weather is not conducive to the bacteria expressing virulence factors and subsequently causing lesions [11]. In this way, infection can spread undetected, and may not cause disease under suboptimal conditions, with the virulence of the infecting strain potentially masked. The presence of a virulent strain does not guarantee the development of virulent footrot [12, 13], but detection may be useful as an indication of clinical disease risk. In addition, footrot is a highly contagious disease, with spread of the bacteria occurring easily through pasture and yards, and the presence of asymptomatic carriers commonplace [14].

Laboratory based diagnostic tools, such as the gelatin gel, elastase and the *intA* polymerase chain reaction (PCR) do not always align with the clinical presentation and severity of disease and use of these assays is the cause of much debate [15–17]. A more informative approach is to use a laboratory-based footrot diagnostic test that can detect infection and identify the virulence potential of the infecting strain early in the disease. A recently published real-time PCR able to detect and differentiate *aprV2* and *aprB2* provides this information [5].

An extension of this diagnostic capability is to test on farm for infection, with a molecular assay that can be performed in a shearing shed or paddock, alongside other every day management practices. Fast identification of infection and strain typing of *D*. *nodosus* has the potential to improve biosecurity practises, reduce disease spread, and implement timely and cost-effective control methods. Loop mediated isothermal amplification (LAMP) is a nucleic acid amplification method that has been reported to be robust, simple to use and appropriate for application in the field [18]. LAMP reactions consist of four main primers, a forward and backward inner primer (FIP and BIP), and inner (F3) and outer primer (B3). These four primers are designed to recognise 6 distinct sequence regions on the target, with FIP and BIP containing complimentary sequences to form loop structures, and an additional two primers, loop forward (LF) and loop back (LB), can be designed to an additional 2 sequence regions, to form loop structures and amplification starting points, increasing reaction speed [19]. LAMP utilises a unique polymerase with strand displacement activity and as such does not require thermocycling, and lacks proof-reading ability during amplification [20, 21]. LAMP is highly specific for detecting a target sequence within a species or gene, however can be challenging for SNP detection. Commercially available master mixes contain intercalating fluorescent dyes that allow the real-time monitoring of amplification. These properties combine to offer a fast, robust, specific and sensitive assay that uses simple, portable machinery, in field. We report the development of a LAMP that detects *aprV2* in *D*. *nodosus* (VDN LAMP), the assay performance in comparison to a previously published rtPCR and the ability to identify virulent *D*. *nodosus* infections on farm.

## Materials and methods

### Control strains

Genomic DNA (gDNA) was extracted from cultured cells of *D*. *nodosus* strain A198 (*aprV2*) (AC: 6466) and strain C305 (*aprB2*) (AC: 6465) using PrepMan Ultra Sample Preparation (Life Technologies) as per manufacturer’s instructions. Genomic DNA (gDNA) extracted from these *D*. *nodosus* strains were used as controls throughout this study and were provided by DAFWA Diagnostics and Laboratory Services (Department of Agriculture and Food, South Perth, Western Australia). DNA was quantitated at 260 nm using a Nanodrop (ThermoFisher).

### Sample collection

Samples for rtPCR and VDN LAMP use were obtained from the Victorian Government Veterinary Diagnostic Laboratory [22]. Samples were submitted by Victorian District Veterinary Officers, Animal Health Officers, and private veterinary practitioners during routine disease investigations, from December 2014 to July 2015.

For rtPCR, the interdigital skin or active margin of a lesion was swabbed with sterile cotton swabs (CLASSIQSwabs) and placed into 600 μL of phosphate buffered saline (PBS) (8.1 mM Na_2_HPO_4_^3-^, 137 mM NaCl, 1.4 mM KH_2_HPO_4_ and 2.6 mM KCl) containing 20 mM ethylenediaminetetraacetic acid (EDTA), pH 8. Using the modified Egerton foot scoring system [6] (Table 1) foot scores of the sampled feet were taken and recorded. Samples were stored and transported at 4°C before use.

Animal experimental procedures for collection of field-based VDN LAMP samples were approved by the La Trobe University Animal Ethics Committee (ethics approval AEC17-21). VDN LAMP field swabs were collected as above from August–September 2017 into 500 μL alkaline-polyethylene glycol 200 (Sigma Aldrich), pH 13 [23]. Template was prepared by directly diluting the sample using a 10 μL inoculation loop into 990 μL H_2_O (MilliQ). Method of collection and foot scores were recorded as described. For both rtPCR and field-based VDN LAMP throughout, the highest scored foot (singular) was sampled per sheep.

**Table 1:** Modified Egerton foot scoring system used to class clinical signs of footrot of sampled sheep.

Score	Description
0	Normal foot with no lesion.
1	A limited mild interdigital dermatitis.
2	A more extensive interdigital dermatitis.
3	Severe interdigital dermatitis and under-running of the horn of the heel and sole.
4	Severe interdigital dermatitis and under-running of the horn of the heel and sole but with the under-running extending to the walls of the hoof.
5	Necrotising inflammation of the deeper epidermal layer (laminae) of the abaxial wall with consequent under-running of the hard horn of the hoof.

### DNA extraction

DNA for the specificity panel was extracted using the Power-Soil DNA isolation kit (MOBIO), as per manufacturer’s instructions, with the following modifications at the elution stage: 30 μl of nuclease free water, pre-heated to 30°C, was added to the white filter membrane, followed by an incubation period of five minutes at room temperature.

Nucleic acid for rtPCR and lab based VDN LAMP was extracted and purified from collected samples using the MagMax Viral RNA extraction kit (Thermo Fisher Scientific), and Kingfisher-96 magnetic particle handling system (Thermo Fisher Scientific), as per manufacturer’s instructions.

### *D*. *nodosus* strain typing using rtPCR

The presence of *aprV2* and/or *aprB2* in samples was identified using primers, probes and cycling conditions as described by Stäuble et al. [5]. The AgPath-ID One Step RT-PCR Kit (Ambion) was used as master mix according to manufacturer’s instructions, with primers and probes synthesised and supplied by Applied Biosystems. Reactions were carried out in 25 μL volumes and analysed using the 7500 Fast Real-Time PCR System (Life Technologies), with a set threshold of 0.05. Results are reported as cycling threshold (Ct) values, the point at which the sample signal exceeds the threshold of 0.05.

### Design of LAMP assay primers

LAMP primers were designed using Primer Explorer V5 (Eiken Chemical Company; https://primerexplorer.jp/e/) with the forward inner primer (FIP) and backward inner primer (BIP) targeting the 2-base pair SNP site in *aprV2* (accession number: L38395.1) at position 661–662 (Table 2, S1 Fig). Different combinations of primers, including FIP, BIP, F3, B3, LF and LB were tested and a set of 5 primers consisting of a FIP, BIP, F3, B3 and LF were chosen. The 2bp SNP site (bold) overlaps at the 5’ end of the F1 complementary region contained in FIP and the 5’ end of the B1 region found in BIP, to facilitate discrimination between the *aprV2* and *aprB2*. All primers were assessed for initial specificity using a BLAST search with GenBank.

**Table 2:** Primer sequence and corresponding LAMP primer design region on sequence. Underlined regions indicate sequence is complementary to 5’– 3’ sequence regions.

Primer	Primer Sequence 5’– 3’	Sequence Region 5’ - 3’
FIP^1^	**TA**ACCACCGCATGCCCAGTTATCAAACCAGTCGCAATAGCCAAATTTCTTTAGATGG	F2, F1
BIP^1^	**TA**TCCTGATCCACGCAAAGAAAGAAGCGGTTATTGGTTACCGCAGC	B2, B1
F3^1^	CGTTTTACCAGGTTATGACTT	F3
B3^1^	CACCAGCAACACCGATAC	B3
LF^1^	TCAGCATCGCGACCATCA	LF
LB^2^	ACAGCTCTTGGCACGGTTCAC	LB

^1^ Primer used in final assay.

^2^ Primer not used in final assay.

### Assay optimisation

LAMP reactions were carried out in 25 μL volumes using 15 μL OptiGene GspSSD2.0 Isothermal Mastermix (ISO-DR004), 5 μL primer mix (final concentrations of 1.6 μM FIP and BIP, 0.2 μM F3, B3 and LF) (Bioneer), and 5 μL template. Template included MilliQ nuclease free water as no template control, and control isolate A198 and C305 gDNA. Assays were run on both the Genie II and Genie III (Optigene) real-time fluorometer, with results reported as time to positive (Tp) (minutes.seconds) and anneal derivative melting temperature (Tm) (°C), given when the sample florescence crossed the pre-set threshold of the Genie machines. Primer concentration ratios of 1:10, 1:8, 1:6 and 1:4 (F3/B3:FIP/BIP) were assessed and 1:8 chosen to optimise the discrimination of *aprV*2 fluorescent signals from *aprB2* fluorescent signals based on Tp. Temperature gradients from 60°C—67°C were performed and assessed for speed and discrimination. LAMP assays were run with a pre-heating step of 40°C for 1 minute, followed by 20 minutes at 65°C before an annealing step from 94°C to 84°C, at a rate of 0.5°C/second, with these conditions used throughout.

### Analytical performance of VDN *D*. *nodosus* LAMP

The analytical sensitivity of the VDN LAMP was determined using a standard curve of gDNA from virulent (*aprV2*-positive) and benign (*aprB2-*positive) *D*. *nodosus* as the template with final concentrations of 5, 0.5, 0.05, 0.025 and 0.005 ng μL^-1^. Samples were run in triplicate across both the Genie III real-time fluorometer and the Genie II real-time fluorometer (n = 3, each dilution). Analytical sensitivity of the assay was determined in duplicate on the Genie II real-time fluorometer and repeated singularly on the Genie III real-time fluorometer. It was necessary to run samples in this manner due to machine processing limitations as Genie II can only run 8 samples at one time while Genie III can run 16 samples.

A panel consisting of gDNA from 12 different bacterial species (Table 3) was used to determine specificity, kindly supplied by Dr Ashley Franks, La Trobe University. Additionally, samples processed with the MagMax Viral RNA Extraction kit, negative for *D*. *nodosus* (n = 46), positive for *aprB2* (n = 7) and field prepared negative samples (n = 23) and *aprB2* positive samples (n = 9), were also used to determine specificity of the VDN LAMP.

**Table 3:** Bacteria commonly found in the sheep environment used to demonstrate specificity.

Organism	Source	Strain number
*Bacillus cereus*	University of Queensland	UoQ 446
*Corynebacterium xerosis*	University of Melbourne	UoM 187
*Escherichia coli*	University of Melbourne	UoM 182
*Proteus mirabilis*	University of Queensland	UoQ 21
*Proteus vulgaris*	University of Queensland	UoQ 22
*Pseudomonas aeruginosa*	University of Queensland	UoQ 16
*Salmonella typhimurium*	University of Queensland	UoQ 342
*Shigella sonnei*	University of Queensland	UoQ 158
*Staphylococcus aureus*	University of Queensland	UoQ 111
*Staphylococcus epidermidis*	University of Queensland	UoQ 105
*Staphylococcus epidermidis*	University of Queensland	UoQ 105
*Streptococcus pyogenes*	La Trobe University	LTU 123

Reproducibility of the VDN LAMP was performed using sheep samples (n = 21) extracted and purified by the MagMax Viral RNA Extraction kit. These samples were run in triplicate over 3 different days to assess intra-assay variation, while triplicate *aprV2* gDNA controls across sensitivity concentrations were used to calculate inter-assay variation.

### Comparison of *aprV2/aprB2* rtPCR to VDN LAMP

One hundred and forty-three samples were obtained retrospectively from the Victorian Government Veterinary Diagnostic Laboratory. Sample extraction for rtPCR was as described and rtPCR was performed as previously stated. Samples with a Ct value under 35 are considered positive for *D*. *nodosus*. VDN LAMP was performed as described above on the same nucleic acid extractions for comparison between methods to assess agreement.

### Field performance

The VDN LAMP assay as developed in the laboratory was tested in-field as proof of concept, using the alkaline-PEG sampling method outlined above. Foot swabs were collected from August–September 2017 into 500 μL alkaline-PEG, diluted 1:100 into MilliQ H_2_O, and 5 μL of this dilution used directly as template in-situ on farm. Samples (n = 57) were collected from sheep on 3 different farms (Farm 1: Shire of Strathbogie, Farm 2: Shire of Southern Grampians and Farm 3: Shire of Hepburn), covering the full range of foot scores from 0–5, based on the Egerton foot scoring system (Table 1). Samples typically contained hair, faeces, soil and plant material. Biologically duplicate samples were also collected for screening using the *aprV2/aprB2* rtPCR as described above to compare results.

### Statistics

Statistics were performed using Microsoft Excel 2016 and GraphPad Prism 6. Co-efficient of variation was calculated to indicate repeatability using

$$CV=\frac{SD}{\bar{x}}X\;100$$

The level of agreement between rtPCR and VDN LAMP was evaluated using Cohen’s kappa statistic from Fleiss, Levin [24] and interpreted using the strength of agreements of the Altman scheme where ≤0 = worse than chance alone, <0.20 = poor, 0.21–0.40 = fair, 0.41–0.60 = moderate, 0.61–0.80 = good, and 0.81–0.99 = very good, 1.00 = perfect. To establish if there was a statistically significant difference between the two methods in designating virulence, McNemar’s Chi-Square Test for Paired Observations was used. VDN LAMP diagnostic sensitivity (DSe) is defined as the percentage of VDN LAMP *aprV2* positive samples within *aprV2* positive rtPCR samples, whilst VDN LAMP diagnostic specificity (DSp) is defined as the percentage of VDN LAMP negative samples within *aprB2* positive or *D*. *nodosous* negative samples as designated by rtPCR.

## Results

### Design of LAMP primers and assay optimisation

Initial assessment of the VDN LAMP primer sets was undertaken using high quality gDNA from two previously characterised *D*. *nodosus* isolates, A198, as the *aprV2* control, and C305, as the *aprB2* control. Various primer set combinations and concentrations were assessed for performance (Table 4). The primer combination in bold was chosen due to providing the fastest time to signal detection and the best discriminatory power between *aprV2* and *aprB2*, based on Tps’, using 0.5 ng μL^-1^ genomic DNA as template, and was subsequently used throughout. The recommended temperature for OptiGene GspSSD 2.0 Isothermal Mastermix (ISO-DR004) of 65°C was found to give the fastest time to positive, with changes in temperature (60–67°C) not affecting specificity of the reactions, only the time of amplification.

**Table 4:** The effect of different FIP, BIP, F3, B3, LF and LB primer final concentrations and combinations on amplification time of 0.5 ng μL^-1^ *aprV2*-positive control and ng μL^-1^ *aprB2*-positive control gDNA.

FIP+BIP (μM)	F3+B3 (μM)	LF+LB (μM)	LF (μM)	LB (μM)	*aprV2* Tp	*aprB2* Tp
2	0.2				12.15	-
1.6	0.2				12.45	-
1.6	0.2	0.1			8.3	14.45
1.6	0.2	0.2			8.15	14
1.6	0.2			0.4	8	14
1.6	0.2			0.2	8.45	15
1.6	0.2		0.4		8	14
1.6*	**0.2**		**0.2**		**9.45**	**18**
1.2	0.2				14.15	-
1.2	0.2		0.2		11.3	-
1.2	0.2	0.4			6.15	10.15
0.8	0.2	0.4			7.45	12.15

* Bold type indicates the final primer concentrations of the chosen primer set.

### Analytical performance of VDN LAMP

**Fig 1:**
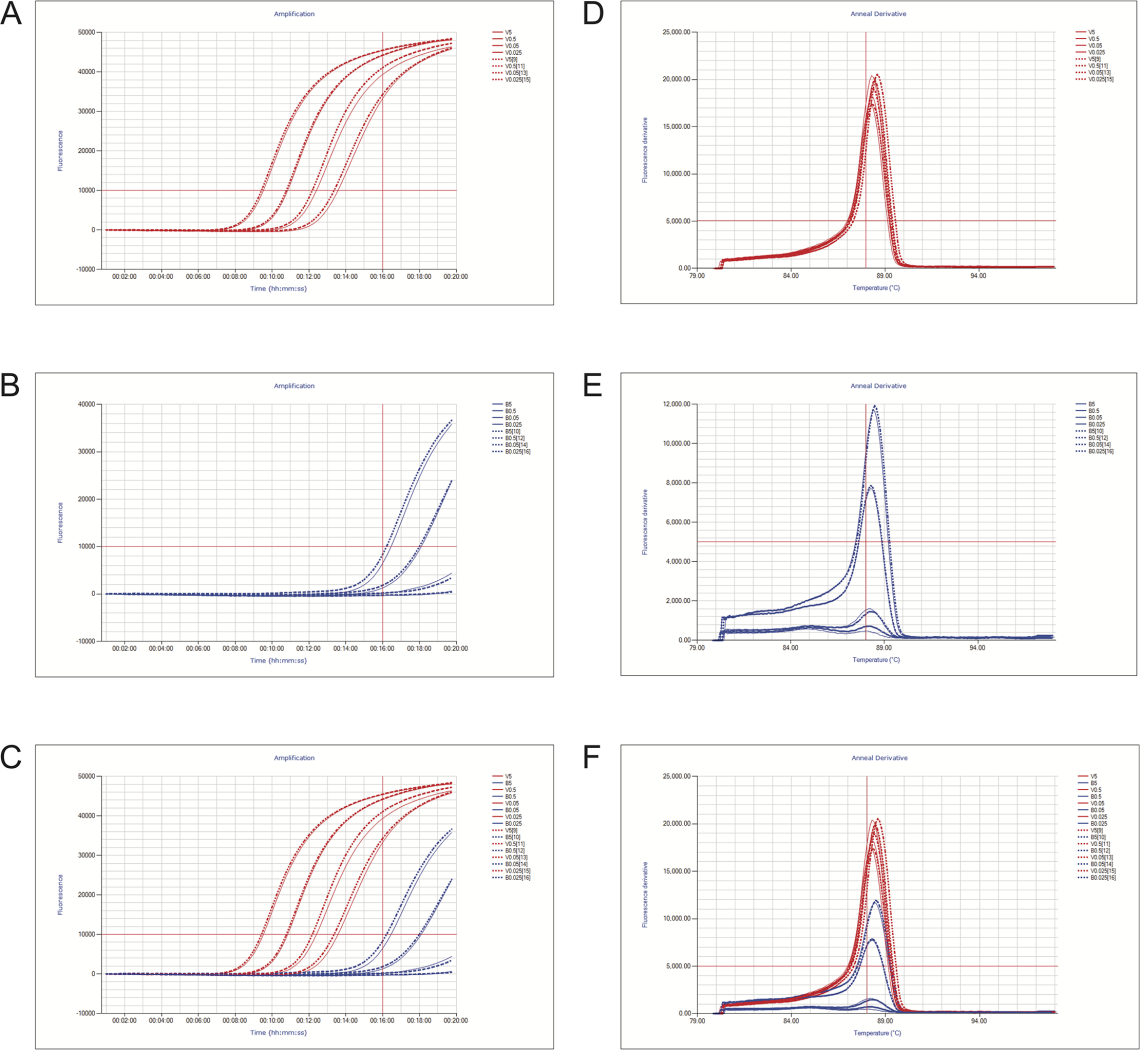
Amplification curves and anneal derivative melting temperatures for *aprV2*. gDNA dilutions and *aprB2* gDNA dilutions from 5–0.025 ng μL^-1^. Amplification curve thresholds for time to positive are indicated in red on graphs A, B and C. A–*aprV2* gDNA dilutions from 5–0.025 ng μL^-1^ amplification; B—*aprB2* gDNA dilutions from 5–0.025 ng μL^-1^ amplification; C–both gDNA dilutions from 5–0.025 ng μL^-1^ amplification. The fluorescent derivative annealing curve for the corresponding *aprV2* gDNA dilutions (D), *aprB2* gDNA dilutions (E), and both (F) are shown with the threshold for peak detection in the anneal derivative and the acceptable range of Tm shown in red. *AprV2* controls are indicated by red and labelled ‘V’, with final concentrations (ng μL^-1^) of gDNA shown numerically following. *AprB2* controls are indicated by blue and labelled ‘B’, with final concentrations (ng μL^-^1) of gDNA shown numerically following. For all, solid lines are the 1^st^ repeat, dashed the 2^nd^.

To assess VDN LAMP sensitivity to ensure adequate detection and discrimination of *aprV2* from *aprB2*, a serial dilution of gDNA for both *aprV2* and *aprB2 D*. *nodosus* isolates was used. Using diluted gDNA at final concentrations of 5, 0.5, 0.05, 0.025 and 0.005 ng μL^-1^, the chosen VDN LAMP primer set amplified *aprV2*-positive gDNA within the range of 5–0.005 ng μL^-1^ reliably between a Tp range of 9.45–15.53 minutes (Fig 1), and with a Tm range of 88.00°C– 88.60°C. Based on these results, a sample that gave a Tp of ≤ 16 minutes was considered positive for the presence of *aprV2*. Very low concentrations of virulent *aprV2*-positive gDNA (0.0005 ng μL^-1^) showed inconsistent amplification after 16 minutes. Genomic DNA from *aprB2*-positive strain was amplified within a range of 5–0.5 ng μL^-1^ (Fig 1), with times of 16.58–18.2 minutes (Table 5) and Tm’s of 88.20°C and 88.50°C. Based on these initial observations, samples that gave a Tp range from 16–20 minutes were subsequently designated as ‘uncertain’, as both very low concentrations of *aprV2*-positive gDNA and very high concentrations of *aprB2*-positive gDNA gave results within this range. Amplification of only *aprV2* or *aprB2* was confirmed by a single Tm peak, seen within the range of 88.0°C– 88.9°C, and was required to be counted as VDN LAMP positive. Samples that gave only a Tp, only a Tm or no Tp and Tm were considered VDN LAMP negative. A run was considered valid if the *aprV2* gDNA control amplified with a Tp and Tm within the stated range, and the negative control had no amplification curve and anneal derivative melting temperature.

**Table 5:** gDNA concentration of control strains and inter-assay co-efficient of variation (CV%) for *D*. *nodosus* gDNA controls using VDN LAMP.

	Virulent (*aprV2*) *D*. *nodosus*	Benign (*aprB2*) *D*. *nodosus*
Final [DNA] (ngμL^-1^)	Average Tp (CV%)	Average Tp (CV%)
5	09.45 (0.00%)	16.58 (2.22%)
0.5	11.15 (0.78%)	18.20 (0.95%)
0.05	12.40 (0.70%)	-
0.025	13.92 (3.10%)	-
0.005	15.53 (6.06%)	-

As the VDN LAMP assay’s intended use is in the field where a variety of environmental bacteria will be present, specificity of the assay was tested. Genomic DNA from range of environmental bacteria listed in Table 3 was tested and no bacteria returned a positive result (S2 Fig), indicating the VDN LAMP assay is specific for *D*. *nodosus*. Additional specificity testing was performed on field collected foot swabs that were shown to be *D*. *nodosus* negative by rtPCR. None of the 46 *D*. *nodosus* negative samples with nucleic acid isolated and purified showed any positive amplification further showing that VDN LAMP is specific for *D*. *nodosus* (S1 Table).

To investigate repeatability of the assay, both control strains and a subset of field samples were used. Field samples with nucleic acid isolation and purification were used. The sample subset was chosen to provide a variety of Ct ranges and *aprV2/aprB2* rtPCR designations. Inter-assay co-efficient of variation (CV) for *aprV2* control Tps’ ranged from 0–6.06%, most to least concentrated (Table 5). *AprB2* controls gave an inter-assay CV range from 2.22–0.95% (Tp) (Table 5). Using the field sample subset, intra- assay CV for individual samples ranged from 1.2–10.25% (Tp) (Table 6). VDN LAMP intra-assay CV generally increased with increasing Ct value (therefore decreasing number of *D*. *nodosus*) of the sample.

**Table 6:** Lab processed field samples with virulence designated by rtPCR and individual sample CV’s.

Sample ID	*aprV2* Ct	*aprB2* Ct	Average Tp (CV%)	Average Tm (CV%)
315	21	-	11.87 (08.27%)	88.43 (0.07%)
318	24	-	12.25 (07.76%)	88.33 (0.07%)
220	24	32	12.87 (08.66%)	88.33 (0.07%)
196	25	-	14.48 (10.25%)	88.27 (0.07%)
2014–2350 4	25	25	12.30 (01.22%)	88.30 (0.11%)
2014–2350 5	27	28	13.25 (01.31%)	88.23 (0.13%)
2014–2350 8	27	26	13.35 (01.30%)	88.23 (0.13%)
13A	28	29	16.10 (06.68%)	88.13 (0.28%)
30	30	-	17.15 (09.43%)	88.27 (0.07%)
2014–2177 6	31	-	-	-
2014–2178 8	31	-	-	-
10A	37	27	-	-
289	-	33	-	-
2014–2183 3	-	23	-	-
2014–2183 6	-	23	-	-
2014–2183 5	-	26	-	-
2014–2183 4	-	24	-	-
305	-	28.4	-	-
6	-	-	-	-
146	-	-	-	-
214	-	-	-	-

### Comparison of *aprV2/aprB2* rtPCR to VDN LAMP

Using 143 lab processed field samples, VDN LAMP was performed on the same nucleic acid extractions as rtPCR to assess VDN LAMP diagnostic sensitivity, specificity and agreement to the *aprV2/B2* rtPCR. From these samples, 90 were rtPCR *aprV2* positive (Ct range 20.40–34.25), 4 samples were co-infected with both *aprV2* (Ct range 24–37) and *aprB2* (Ct range 24.56–39.85), 7 were *aprB2* positive (Ct range 23.4–33), and 46 samples were *D*. *nodosus* negative.

The VDN LAMP identified 75/143 samples as *aprV2* positive, with all 75 also *aprV2* positive by rtPCR (Table 7). For the 53 samples rtPCR *aprB2* positive or *D*. *nodosus* negative, 0/53 were VDN LAMP positive (Table 7). This resulted in the definition of a VDN LAMP positive sample being any sample that gave a Tp ≤ 20 minutes and a corresponding single anneal derivative peak with a Tm of 88.0°C– 88.9°C. It is recommended that samples with Tps’ above 16 minutes are confirmed via rtPCR.

**Table 7:** Comparison of *aprV2* detection between VDN LAMP and *aprV2/aprB2* rtPCR on 143 samples processed using MagMax Viral RNA Extraction kit.

		*aprV2/aprB2 rtPCR*		
		*aprV2* positive	*aprV2* negative	*Total*
**VDN LAMP**	**Positive**	75	0	*75*
	**Negative**	15	53	*68*
	*Total*	*90*	*53*	*143*

McNemars X^2^ = 13.067, *P =* 0.0003; kappa statistic = 0.788, 95% CI 0.688 to 0.887, ‘good’ agreement

DSe = 83.33% (95% CI 74.00 to 90.36) DSp = 100.00% (95% CI 93.28 to 100.00)

The VDN LAMP gives a DSe of 83.33%, and DSp of 100%, showing ‘good’ agreement (κ = 0.788) to the rtPCR virulence designation.

VDN LAMP decreased in sensitivity with an increase in the samples Ct value from *aprV2/aprB2* rtPCR (Table 8). Of the 13 samples with Ct values 30–35, 3 were identified as *aprV2* positive by the VDN LAMP. The number of samples VDN LAMP correctly identified as *aprV2* positive increased as the Ct value lowers, corresponding with an increasing number of bacteria present as indicated by the rtPCR. For those samples under a Ct of 25, 39/40 were correctly identified by the VDN LAMP, and 33/37 within the Ct range of 25–29.

**Table 8:** The number of samples correctly identified as virulent by VDN LAMP in different Ct ranges from those samples positive for *aprV2* via rtPCR.

Ct range	Number of samples	VDN LAMP positive	DSe (%)
< 25	40	39	97.50
25 < 30	37	33	89.19
30 < 35	13	3	23.08

### Field performance

To trial the field application of the VDN LAMP, 57 samples were collected over 3 volunteer farms. For a sample to be considered positive in the field, both a time to positive (Tp) and a single peak melting temperature (Tm), as previously defined, was required. A run was considered valid as defined previously. Foot scores, VDN LAMP, rtPCR results, and individual farm agreements between methods are summarised (Table 9).

**Table 9:** The foot scores of sampled sheep, VDN LAMP and rtPCR *aprV2* positive samples and subsequent agreement within each farm.

Farm #	Score (number of animals)	VDN LAMP+^1^	rtPCR *aprV2*+	Agreement (%)
1	0 (29), 1 (1)	1	1	100
2	0 (1), 1 (10), 2 (3)	13	14	92.86
3	0 (6), 1(2), 2(1), 5 (4)	8	9	88.89

^1^VDN LAMP positive samples were obtained only on those samples also rtPCR *aprV2* positive

From farm 1, 1/30 samples returned a VDN LAMP Tp of 15.45 minutes, and a Tm of 88.14°C. This sample was positive for *aprV2* and *aprB2* using rtPCR, while an additional 9 samples were *aprB2* positive with rtPCR, and VDN LAMP negative. 20/30 samples were *aprV2* or *aprB2* negative on both VDN LAMP and rtPCR.

Within farm 2, 13/14 were VDN LAMP positive, with Tp’s ranging from 13.13–19.15 minutes and Tm’s from 88.16–88.36°C. From this group, 14/14 were rtPCR *aprV2* positive.

Farm 3 had 8/13 samples VDN LAMP positive, with Tp’s from 13.45–18 minutes, and Tm’s of 88.06–88.46°. The 8 VND LAMP samples were all also *aprV2* positive from rtPCR. An additional 2/13 samples were rtPCR positive for *aprV2*, and VDN LAMP negative, with 1 sample discounted as rtPCR positive for *aprV2* after the Ct cut off of 35 is applied, with VDN LAMP then correctly identifying 8/9 samples as *aprV2* positive A total of 3 samples were both VDN LAMP and rtPCR negative for *aprV2* and *aprB2*. Farm 3 had the highest proportion of severe lesions as shown by foot score.

The pilot field study gave an overall VDN LAMP DSe of 91.67% (22/24), and DSp of 100% with no *aprB2* positive (n = 9), or *D*. *nodosus* negative (n = 24) samples giving a positive VDN LAMP result (Table 10). These results suggest the VDN LAMP assay can identify virulent *D*. *nodosus* infections in the field.

**Table 10:** Comparison of *aprV2* detection between in-field VDN LAMP and *aprV2/aprB2* rtPCR on 57 field processed samples.

		*aprV2/aprB2* rtPCR		
		*aprV2* positive	*aprV2* negative	*Total*
**VDN LAMP**	**Positive**	22	0	*22*
	**Negative**	2	33	*35*
	*Total*	*24*	*33*	*57*

McNemars X^2^ = 0.500, *P =* 0.4795; kappa statistic = 0.927, 95% CI 0.828 to 1.000, ‘very good’ agreement

DSe = 91.67% (95% CI 73.00 to 98.97) DSp = 100.00% (95% CI 89.42 to 100.00)

## Discussion

Footrot is a contagious disease that is easily spread throughout the farm. Traditionally, visual inspections are used to make a clinical diagnosis, with laboratory support occasionally used. Laboratory methods previously available for a diagnosis are time consuming and require specialist growth media and expertise. The improved ability to detect virulent *D*. *nodosus* prior to the appearance of severe clinical symptoms can help inform control methods on-farm and reduce the severity of disease if treated early. LAMP assays have been developed for several livestock diseases [25–27] and are reported to offer a field suitable, easily performed molecular diagnostic assay for detection of infection. While many livestock LAMP assays have been reported, very few have progressed to field testing and demonstrated on-farm use [28, 29] with most in-field LAMP testing occurring in plant diagnostics [30, 31]. Here, we present a LAMP assay developed in the laboratory and applied in-field.

The VDN LAMP assay with 5 primers that is presented in this paper is based on 7 regions of the *aprV2* gene sequence found in *D*. *nodosus*, only one of which is differing between *aprV2* and *aprB2*. As the primers have multiple shared sequence between virulent and benign *D*. *nodosus*, the FIP and BIP primers were used for their discriminatory power. This strategy has been used for SNP detection previously [32]. To help with differentiation, the FIP and BIP primers were designed to overlap at the SNP site, on the 5’ end of each which was found to be most effective. The use of 5 primers in a LAMP assay has also been reported previously, to detect *Ralstonia solanacearum* [33]. Traditional methods of improving oligo binding stringency, like temperature adjustments, were seen here to adversely affect the amplification of the target. Due to this, the 5 primer set was chosen as it offered the best differentiation between *aprB2* and *aprV2* on high quality gDNA when based on Tp and also amplified a wide concentration range of *aprV2* DNA consistently within the 20 minute run time. The *aprV2* gene was chosen as the target due to the potential for severe lesion development, and the cost of control programs once disease progresses through to virulent clinical signs.

The original aim of the assay was to differentiate samples in the field with *aprV2* present from those with benign *D*. *nodsous* (*aprB2*) infections. Although the VDN LAMP assay amplified *aprB2* gDNA, it was a noticeably slower reaction than *aprV2*, only amplifying after 16 minutes and only when at high concentrations. Amplification of *aprB2* from gDNA was 2 orders of magnitudes less than that of *aprV2*, with no concentration below 0.5 ngμL^-1^ showing positive results, in comparison to 0.005 ngμL^-1^ of virulent *D*. *nodosus* gDNA. It is recommended that samples amplifying after 16 minutes have the presence of *aprV2* confirmed via rtPCR, based on gDNA laboratory results. It was noted that when used on field sample template with nucleic acid isolated and purified, samples rtPCR positive for a range of *aprB2* Ct values were not VDN LAMP positive. It’s thought the addition of remaining inhibitors in the extraction to the already poor amplification of *aprB2* is responsible for hindering the reaction and the cessation of *aprB2* amplification. No rtPCR *aprB2* positive samples, both nucleic acid isolated and purified or field prepared, showed a positive VDN LAMP result. This continued observation supports the VDN LAMP only amplifying samples which have *aprV2* present. For both the nucleic acid isolated and purified samples and the field processed data, diagnostic specificity of the VDN LAMP assay was 100%.

The sensitivity of VDN LAMP was less than that of rtPCR, which has been reported previously [34]. VDN LAMP DSe varied from 97.5–23.08%, depending on the samples Ct value, and therefore the number of bacteria collected on the swab (Table 8). The diagnostic sensitivity observed indicates the in-field application of VDN LAMP use may lie at the flock level, with this being appropriate as typically sheep management decisions are applied at this level. It has been shown that the highest bacterial load occurs in the early stages of disease [35, 36], and this is the concentration range of bacteria where VDN LAMP is most accurate. This suggests VDN LAMP use would be most beneficial at the early stages of clinical disease, to identify if *D*. *nodosus aprV2* is present and provide information that can be acted upon before severe lesions are present.

As a proof of concept for on farm use, the VDN LAMP was deployed on 3 farms with different foot scoring ranges. In all cases, samples were collected and processed on farm with minimal equipment. Reagents were transported in an cold box; a pipette was used to deliver reagents and all buffers and dilutions were carried out in microfuge tubes. A VDN LAMP run was considered valid if the positive and no template H_2_O control performed as expected, and samples were considered positive if a Tp before 20 minutes and a Tm between 88.0–88.9°C was seen, with samples amplifying after 16 minutes confirmed via rtPCR as having *aprV2* present. No runs failed and the average time on farm for collecting and processing 14 samples was 1.5 hours. Samples positive for *aprV2* were detected on all 3 farms. In the case of farm 1, there was 1 instance of co-infection with virulent and benign *D*. *nodosus* as confirmed by rtPCR that was identified as *aprV2* positive by the VDN LAMP, while 9 samples rtPCR positive for only *aprB2* were VDN LAMP negative. The VDN LAMP missed 1 *aprV2* positive sample on farm 2, with this sample giving a positive result that was later discounted as the Tm (87.86°C) did not meet the quality control requirements of a positive sample. Farm 3, which had been recently diagnosed by a private veterinarian as having virulent footrot, had 8/9 samples rtPCR *aprV2* positive samples correctly identified by the VDN LAMP. One *aprV2* rtPCR positive sample was missed by the VDN LAMP. The remaining 4/13 samples from farm 3 were *aprV2* negative on both the VDN LAMP and rtPCR.

The in-field VDN LAMP assay was able to correctly identify 55/57 (96.49%) samples when compared to rtPCR, without requiring any type of sample nucleic acid isolation or purification. The LAMP platform has been shown to perform when large amounts of inhibitors are present, and in non-sterile or laboratory conditions [37]. The sample substrate commonly found in the pilot study of 57 field samples included soil, faeces, plant material and wool. The field trial of VND LAMP has demonstrated on-farm results and has differentiated *aprV2* from *aprB2* with DSe of 91.67% and DSp of 100%, while being capable of being performed easily and consistently on-farm with minimal equipment. The development of this assay will facilitate changes in approach to footrot control by providing a fast and accurate diagnosis of infection on-farm.

## Conclusion

We have developed a highly specific and sensitive LAMP assay for the detection of virulent *D*. *nodosus*, which was able to correctly identify the presence of the *aprV2* gene in samples collected on swabs in both laboratory and field settings. In the future we envisage the use of the VDN LAMP assay on farm, with continuing improvement to the sample preparation methods and the use of lyophilized reagents.

## Supporting information

S1 Fig LAMP primer set targeting the 2-bp difference of the *aprV2* gene of *Dichelobacter nododus*. (JPG)

S2 Fig Amplification and anneal derivative graphs of specificity panel bacteria presented in Table 3. Bacteria are listed in key and curves correspond to colour. All samples were assayed using the same VDN LAMP primer batch and OptiGene GspSSD2.0 Isothermal Mastermix (ISO-DR004) batch, with conditions as described, on the Genie III fluorometer. A, B–amplification and anneal derivative of *Bacillus cereus*, *Corynebacterium xerosis*, *Escherichia coli*, *Proteus mirabilis*, *Proteus vulgaris Pseudomonas aeruginosa*, *Salmonella typhimurium* and *Shigella sonnei*. C, D–amplification and anneal derivative of *Staphylococcus aureus*, *Staphylococcus epidermidis*, *Staphylococcus epidermidis*, *Streptococcus pyogenes*, negative extraction control, A198 (*aprV2*) gDNA positive control, and no template control. Amplification and corresponding anneal derivative is only seen in the A198 (*aprV2*) gDNA positive control.
(TIFF)

S1 Table Summary table of results for 143 field samples with nucleic acid isolation and purification. (DOCX)
