# Supplementary figures and images for: The development and deployment of a field-based loop mediated isothermal amplification assay for virulent Dichelobacter nodosus detection on Australian sheep
Source: PLoS One. 2018 Sep 27;13(9):e0204310. doi: 10.1371/journal.pone.0204310 (PMC6160043; doi:10.1371/journal.pone.0204310)

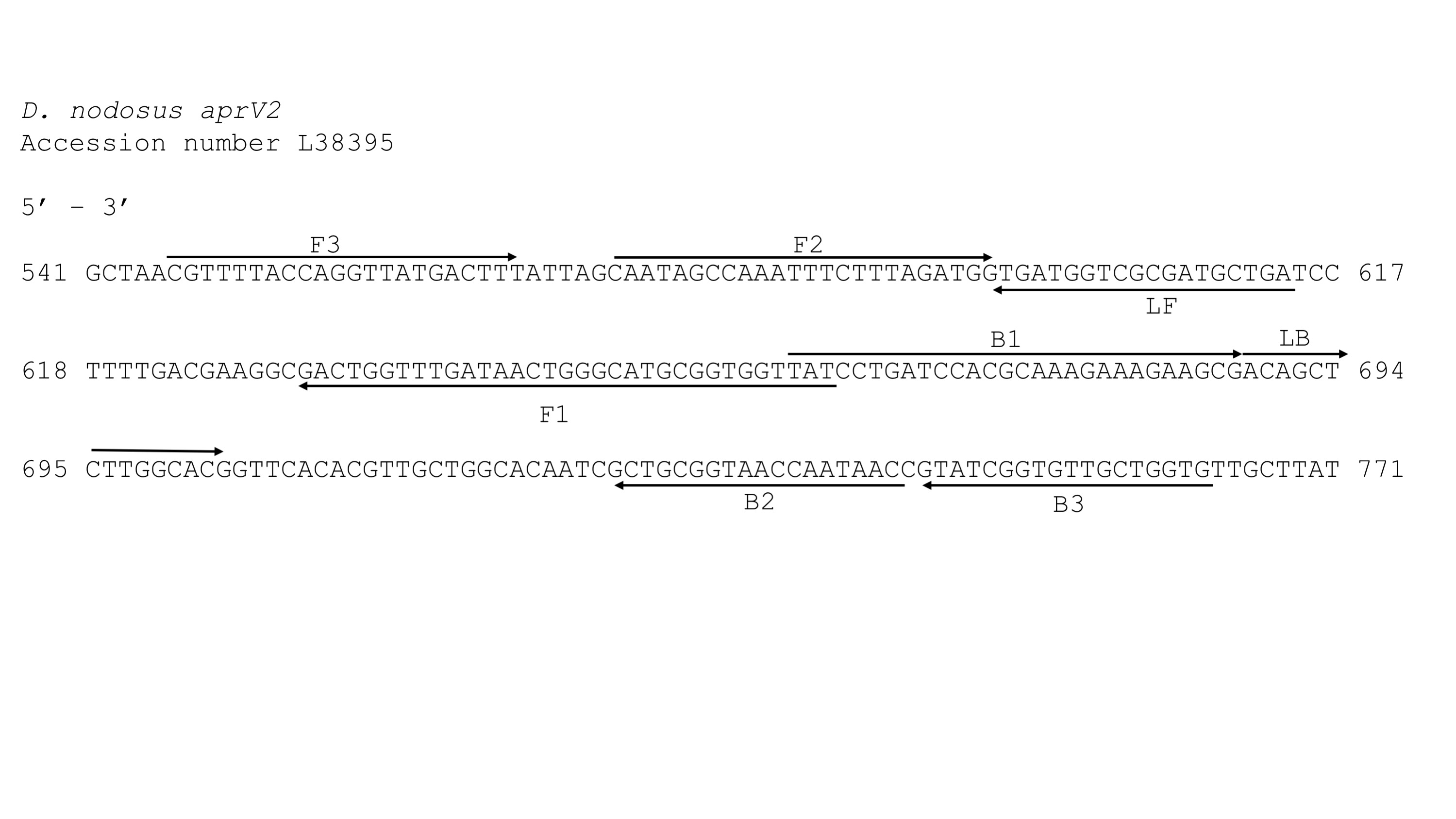

Supplement: S1 Fig — (JPG) [file pone.0204310.s001.jpg]

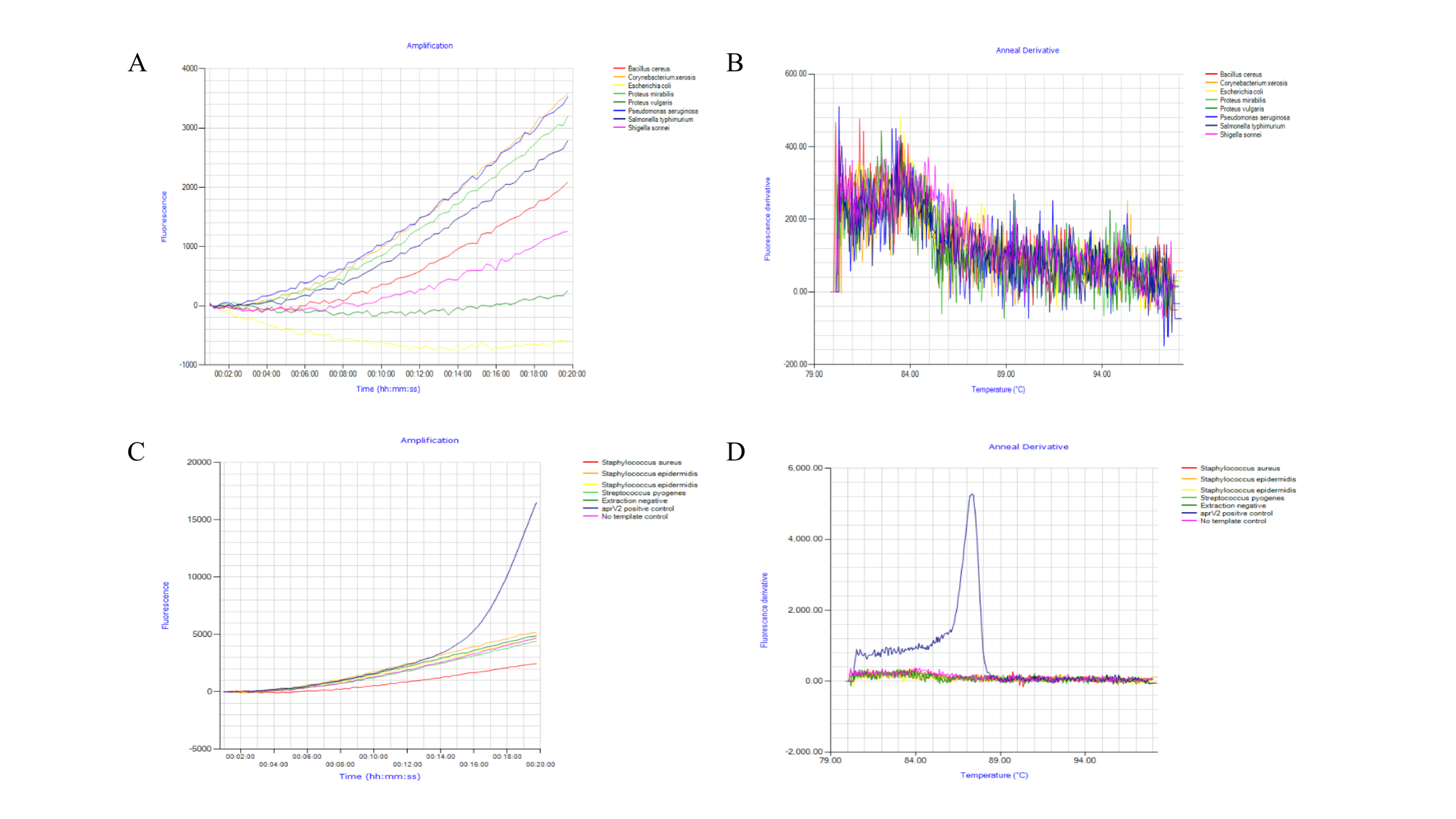

Supplement: S2 Fig — Bacteria are listed in key and curves correspond to colour. All samples were assayed using the same VDN LAMP primer batch and OptiGene GspSSD2.0 Isothermal Mastermix (ISO-DR004) batch, with conditions as described, on the Genie III fluorometer. A, B–amplification and anneal derivative of Bacillus cereus, Corynebacterium xerosis, Escherichia coli, Proteus mirabilis, Proteus vulgaris Pseudomonas aeruginosa, Salmonella typhimurium and Shigella sonnei. C, D–amplification and anneal derivative of Staphylococcus aureus, Staphylococcus epidermidis, Staphylococcus epidermidis, Streptococcus pyogenes, negative extraction control, A198 (aprV2) gDNA positive control, and no template control. Amplification and corresponding anneal derivative is only seen in the A198 (aprV2) gDNA positive control. (TIFF) [file pone.0204310.s002.tiff]
