# Supplementary material for: The development and deployment of a field-based loop mediated isothermal amplification assay for virulent Dichelobacter nodosus detection on Australian sheep
Source: PLoS One. 2018 Sep 27;13(9):e0204310. doi: 10.1371/journal.pone.0204310 (PMC6160043; doi:10.1371/journal.pone.0204310)
Supplement: S1 Table — (DOCX) [file pone.0204310.s003.docx]

S1 Table

Summary table of results for 143 field samples with nucleic acid isolation and purification.

| **Sample** | **Foot score** | ***aprV2* Ct** | ***aprB2* Ct** | **VDN LAMP Tp** |
| --- | --- | --- | --- | --- |
| 6 | 0 |  |  |  |
| 30 | 0 | 30 |  | 19 |
| 33 | 0 | 34.06 |  |  |
| 39 | 2 |  |  |  |
| 63 | 0 |  |  |  |
| 69 | 2 |  |  |  |
| 71 | 0 |  |  |  |
| 72 | 0 |  |  |  |
| 78 | 0 |  |  |  |
| 80 | 0 |  |  |  |
| 93 | 5 | 25.97 |  | 13.3 |
| 94 | 2 | 25.19 |  | 13 |
| 95 | 0 |  |  |  |
| 96 | 1 |  |  |  |
| 97 | 0 |  |  |  |
| 98 | 0 |  |  |  |
| 99 | 0 |  |  |  |
| 100 | 0 |  |  |  |
| 141 | 0 |  |  |  |
| 143 | 0 |  |  |  |
| 146 | 0 |  |  |  |
| 148 | 0 |  |  |  |
| 160 | 0 |  |  |  |
| 171 | 0 |  |  |  |
| 190 | 5 | 33.60 |  |  |
| 193 | 5 |  |  |  |
| 195 | 0 |  |  |  |
| 196 | 5 | 25 |  | 16.15 |
| 199 | 1 |  |  |  |
| 201 | 0 |  |  |  |
| 211 | 0 |  |  |  |
| 214 | 0 |  |  |  |
| 218 | 1 | 24.06 | 39.85 | 12 |
| 219 | 0 |  |  |  |
| 220 | 1 | 24 | 32 | 14.15 |
| 223 | 0 |  |  |  |
| 249 | 0 |  |  |  |
| 282 | 0 |  |  |  |
| 289 | 0 |  | 33 |  |
| 296 | 0 |  |  |  |
| 302 | 0 |  |  |  |
| 303 | 0 |  |  |  |
| 305 | 0 |  | 28.4 |  |
| 306 | 1 | 24.68 |  | 12.15 |
| 307 | 1 | 21.38 |  | 10 |
| 308 | 1 | 30.85 |  | 18 |
| 309 | 0 | 25.68 |  | 13.15 |
| 310 | 0 | 32.41 |  |  |
| 311 | 1 | 22.74 |  | 12.3 |
| 312 | 1 | 24.55 |  | 12 |
| 313 | 0 | 24.92 |  | 12.3 |
| 314 | 0 | 24.89 |  | 13.15 |
| 315 | 1 | 21 |  | 13 |
| 315 | 1 | 21.50 |  | 12 |
| 316 | 0 | 21.80 |  | 11.3 |
| 317 | 0 | 24.77 |  | 14.15 |
| 318 | 0 | 22.12 |  | 11.3 |
| 319 | 1 | 24.35 |  | 12 |
| 320 | 0 | 30.65 |  |  |
| 321 | 1 | 23.13 |  | 12.45 |
| 322 | 0 | 26.24 |  | 12.3 |
| 323 | 1 | 24.69 |  | 12.3 |
| 324 | 0 | 23.93 |  | 13 |
| 325 | 0 | 24.61 |  | 12.45 |
| 326 | 0 |  |  |  |
| 327 | 1 | 25.34 |  | 14.45 |
| 328 | 1 | 21.63 |  | 14.3 |
| 329 | 1 | 27.39 |  | 11.3 |
| 329 | 1 | 27.39 |  | 16.15 |
| 330 | 0 | 23.50 |  | 11.45 |
| 330 | 0 | 23.50 |  | 12.15 |
| 331 | 0 | 24.04 |  | 11.3 |
| 331 | 0 | 24.04 |  |  |
| 10A | 1 |  | 27 |  |
| 13A | 1 | 28 | 29 | 16.15 |
| 1B | 0 |  |  |  |
| 2014-2177-1 | 5 |  |  |  |
| 2014-2177-2 | 2 | 24.18 |  | 15 |
| 2014-2177-3 | 5 | 26.36 |  | 14.45 |
| 2014-2177-4 | 5 | 34.25 |  |  |
| 2014-2177-5 | 5 | 26.12 |  | 18 |
| 2014-2177-6 | 5 | 31.69 |  |  |
| 2014-2177-7 | 4 | 26.16 |  | 16.3 |
| 2014-2177-8 | 5 | 25.81 |  | 17.15 |
| 2014-2178-1 | 3 | 29.96 |  |  |
| 2014-2178-2 | 4 | 31.42 |  |  |
| 2014-2178-3 | 3 | 25.32 |  | 14.45 |
| 2014-2178-4 | 2 | 20.4 |  | 13 |
| 2014-2178-5 | 2 | 28.34 |  | 18 |
| 2014-2178-6 | 2 | 24.97 |  | 16.45 |
| 2014-2178-7 | 3 | 25.74 |  | 15.45 |
| 2014-2178-8 | 4 | 31.11 |  |  |
| 2014-2183-1 | 4 | 27.42 |  | 15.45 |
| 2014-2183-2 | 2 | 24.03 |  | 13.45 |
| 2014-2183-3 | 2 |  | 23.48 |  |
| 2014-2183-4 | 4 |  | 24.79 |  |
| 2014-2183-5 | 4 |  | 26.25 |  |
| 2014-2183-6 | 3 |  | 23.57 |  |
| 2014-2183-7 | 3 | 26.39 |  | 16.15 |
| 2014-2183-8 | 2 | 21.62 |  | 14 |
| 2014-2234-1 | 1 | 31.1 |  |  |
| 2014-2234-2 | 1 | 27.02 |  | 18 |
| 2014-2234-3 | 1 | 23.9 |  | 16.45 |
| 2014-2234-4 | 1 | 28.33 |  |  |
| 2014-2234-5 | 1 | 26.61 |  |  |
| 2014-2234-6 | 1 | 27.49 |  | 19.3 |
| 2014-2234-7 | 1 | 28.99 |  |  |
| 2014-2234-8 | 1 | 26.68 |  | 15.3 |
| 2014-2349-2 | 5 | 23.92 |  | 11.45 |
| 2014-2349-3 | 5 | 26.85 |  | 13 |
| 2014-2349-4 | 5 | 27.51 |  | 16.15 |
| 2014-2349-5 | 5 | 25.64 |  | 12.45 |
| 2014-2349-6 | 5 | 27.44 |  | 14 |
| 2014-2349-7 | 5 | 31.64 |  |  |
| 2014-2349-8 | 5 | 26.16 |  | 13 |
| 2014-2349-9 | 5 | 26.52 |  | 12 |
| 2014-2423-1 | 1 |  |  |  |
| 2014-2423-2 | 1 |  |  |  |
| 2014-2423-3 | 1 |  |  |  |
| 2014-2423-4 | 1 |  |  |  |
| 2014-2423-5 | 1 |  |  |  |
| 2014-2423-6 | 1 |  |  |  |
| 2014-2423-7 | 1 |  |  |  |
| 2014-2423-8 | 1 | 25.92 | 24.64 | 13.3 |
| 2014-2436-1 | 3a | 24.73 |  | 12.3 |
| 2014-2436-2 | 4 | 23.62 |  | 12.45 |
| 2014-2436-3 | 3a | 25.95 |  | 13.3 |
| 2014-2436-4 | 3b | 24.6 |  | 13.15 |
| 2014-2436-5 | 4 | 23.88 |  | 11.3 |
| 2014-2436-6 | 3a | 24.4 |  | 12.3 |
| 2014-2436-7 | 2 | 24.52 |  | 12.3 |
| 2014-2436-8 | 3 | 26.78 |  | 13.45 |
| 2014-2519 -1 | 4 | 24.04 |  | 12.45 |
| 2014-2519 -2 | 4 | 29.69 |  | 14.45 |
| 2014-2519 -3 | 4 | 30.23 |  | 17.45 |
| 2014-2519 -4 | 4 | 27.74 |  | 15.3 |
| 2014-2519 -5 | 4 | 23.98 |  | 12.45 |
| 2014-2519 -6 | 4 | 20.94 |  | 11.45 |
| 2014-2519 -7 | 4 | 23.73 |  | 13 |
| 2014-2519 -8 | 4 | 28.42 |  | 14.15 |
| 2B | 0 |  |  |  |
| 3B | 0 |  |  |  |
| 4B | 0 |  |  |  |
